# Shifting dysmenorrhea management from treatment to prevention: the critical adolescent window

**DOI:** 10.3389/fpubh.2026.1812246

**Published:** 2026-05-19

**Authors:** Jiao Wang, Ye Guo

**Affiliations:** 1 Alhambra Medical University, Alhambra, CA, United States; 2 Pain Department, Chengdu Third People's Hospital, Chengdu, China

**Keywords:** adolescence, dysmenorrhea, management, prevention, treatment

## 1 Introduction

Dysmenorrhea is among the most prevalent gynecological symptoms in women, categorized into primary dysmenorrhea, defined by the absence of pelvic organic lesions, and secondary dysmenorrhea, which arises from underlying pathologies including endometriosis and adenomyosis. Primary dysmenorrhea has a particularly high prevalence among adolescent females (1). Epidemiological data indicate that the prevalence of primary dysmenorrhea in adolescent females ranges from 50%−90%, with 15%−25% of affected individuals reporting moderate to severe pain (2, 3). Such pain not only significantly impairs academic performance, social engagement, and activities of daily living, but also increases the risk of developing emotional disturbances including anxiety and depression. However, dysmenorrhea has long been widely dismissed as a normal physiological occurrence in women, with clinical and public health strategies focusing predominantly on symptomatic pain relief, while its long-term health risks and preventability have been largely overlooked. A growing body of cutting-edge research has established that adolescence represents a critical window for both the pathogenesis of dysmenorrhea and the maturation of pain processing pathways (4). Uncontrolled, recurrent menstrual pain can induce central and peripheral pain sensitization, drive the progression of dysmenorrhea from physiological acute pain to chronic persistent pain, and may even act as an independent risk factor for long-term adverse reproductive health outcomes, including endometriosis, chronic pelvic pain, and infertility (5). Building on these findings, this study proposes that adolescence should be recognized as the critical window for dysmenorrhea intervention. Systematic dysmenorrhea screening should be integrated into routine adolescent health examinations and school-based reproductive health education curricula. Through early screening, targeted health education, and timely intervention, we advocate for a fundamental paradigm shift in the management of dysmenorrhea among females, from reactive symptomatic treatment to proactive preventive care.

## 2 Scientific rationale for adolescence as the core window for dysmenorrhea prevention and control

From a pathophysiological standpoint, adolescence is the critical period for the establishment, stabilization, and maturation of the hypothalamic-pituitary-ovarian (HPO) axis. In the 1–2 years following menarche, coinciding with the establishment of regular ovulatory cycles, aberrant endometrial prostaglandin synthesis and release emerges as the core pathogenic mechanism underlying primary dysmenorrhea (6). Concurrently, this period is also critical for the development and maturation of human pain conduction pathways and the central pain regulatory network, which is characterized by marked neural plasticity during this developmental window. Cutting-edge neuroimaging research has demonstrated that recurrent acute menstrual pain stimuli can sensitize nociceptors in the spinal dorsal horn of adolescent females, triggering functional remodeling of central pain regulatory circuits, progressive reductions in pain threshold, and the ultimate establishment of a vicious cycle of pain, sensitization, and further pain exacerbation. Compared with adult women, pain sensitization develops more rapidly in adolescence and exhibits greater reversibility (7). Timely intervention during this window can effectively halt the pathological progression to chronic pain.

Recent pathological and clinical investigations have confirmed that the pathogenesis of endometriosis in approximately 40% to 60% of reproductive-age patients can be traced back to adolescence (8, 9). Progressive dysmenorrhea with onset shortly after menarche represents the earliest clinical manifestation of endometriosis. Owing to the incomplete development of pelvic organs during adolescence, imaging modalities exhibit a low positive detection rate for early-stage diseases. This, together with insufficient clinical awareness in practice, leads to misdiagnosis in the vast majority of affected patients (10). Diagnosis is typically not made until the reproductive years, when patients present with infertility or severe pelvic pain, by which point the optimal window for early intervention has passed.

While dysmenorrhea is often a symptom of endometriosis, early pain management may mitigate neuroinflammatory pathways that contribute to pain chronification, potentially improving long-term outcomes regardless of underlying etiology.

From the standpoint of health behavior establishment, adolescence is a pivotal period for the development of reproductive health literacy and health-related behavioral habits. Health education and intervention delivered during this window can effectively mitigate menstrual-related stigma and correct cognitive misconceptions surrounding dysmenorrhea, while facilitating the establishment of evidence-based pain management and reproductive health self-care practices. The associated health benefits extend across the entire reproductive lifespan and even the full life course, an effect that cannot be replicated by passive interventions initiated in adulthood (11).

## 3 Establishment of an educational and preventive framework centered on the adolescent window

(1) Integration of Standardized Dysmenorrhea Screening into Routine Adolescent Health Examinations and Establishment of a Standardized Screening-Referral Closed-Loop Care Pathway. Globally, and particularly in low- and middle-income countries, reproductive health-related content is broadly and structurally absent from routine adolescent health examination systems, and dysmenorrhea is rarely included as a routine screening indicator (12). This gap results in the vast majority of affected adolescents not receiving early identification and standardized management. From the perspectives of public health policy formulation and adolescent health management standardization, it is imperative to advocate for the inclusion of standardized dysmenorrhea screening as a mandatory module in annual adolescent health examinations. Screening protocols should encompass dysmenorrhea episode frequency, Visual Analog Scale (VAS) pain scores, impairment of academic performance and activities of daily living, associated symptoms, analgesic use history, family history, and red flags for secondary dysmenorrhea (including progressive dysmenorrhea, non-menstrual pelvic pain, and abnormal uterine bleeding). We recommend validated, adolescent-friendly tools such as the Visual Analog Scale (VAS) with a cutoff ≥4/10 for moderate-severe pain, supplemented by the Menstrual Pain Questionnaire (MPQ) to assess functional impairment. Concurrently, a two-tiered “campus initial screening–adolescent gynecology specialist referral” care model should be established. Under this model, adolescents with moderate to severe dysmenorrhea or red flags for secondary dysmenorrhea identified on initial screening are promptly referred to specialist outpatient clinics for standardized assessment and intervention, to minimize missed diagnoses and delayed treatment of underlying organic lesions. School-based screening must prioritize adolescent privacy through confidential self-administered questionnaires, opt-out consent processes, and direct referral pathways that bypass non-essential school personnel to protect student dignity.

(2) Integration of Evidence-Based Dysmenorrhea Management into School-Based Reproductive Health Education Systems to Address Cognitive Barriers and Menstrual Stigma. At present, school-based reproductive health education in most countries worldwide focuses predominantly on reproductive physiological anatomy, sexual health protection, and adolescent psychological adjustment, with very few curricula incorporating content on the evidence-based understanding and standardized management of dysmenorrhea. This gap has contributed to the prevalence of harmful misconceptions among adolescents, including the beliefs that “dysmenorrhea is a normal physiological event that must be endured” and that “analgesic use leads to addiction” (13). These misconceptions, compounded by pervasive stigma surrounding menstrual health topics, result in the vast majority of adolescents with moderate to severe dysmenorrhea failing to actively seek professional medical care (14). It is imperative to systematically integrate dysmenorrhea-related content into the core curriculum of school-based reproductive health education, with tiered instruction tailored to adolescent developmental stages: the early stage (around menarche) focuses on menstrual physiology, hygiene, and the recognition of normal menstrual discomfort; the middle stage covers the pathogenesis of primary dysmenorrhea, evidence-based coping strategies, non-pharmacological interventions, and the safety profile of standardized analgesic use; the late stage supplements this content with red flags for secondary dysmenorrhea, indications for medical evaluation, and the association between dysmenorrhea and long-term reproductive health outcomes. Concurrently, multifaceted approaches including peer education and family-focused educational sessions should be implemented to address societal cognitive biases and foster a stigma-free, help-seeking friendly environment for adolescents.

## 4 Practical challenges and optimization strategies for the paradigm shift to preventive control

The transition from symptomatic treatment to preventive control faces multiple substantial practical challenges: (1) Insufficient societal and professional awareness. Most parents, educators, and even primary healthcare providers continue to view dysmenorrhea as a normal physiological occurrence, with inadequate understanding of its long-term health risks. This knowledge gap hinders the development of a broad social consensus to advance preventive and control initiatives; (2) Gaps in healthcare service systems and limited adolescent gynecology specialty resources. School health providers and maternal and child health personnel lack systematic training in the standardized assessment and management of adolescent dysmenorrhea, creating substantial barriers to the implementation of a functional screening-referral closed-loop pathway; (3) Pervasive menstrual stigma among adolescents. Deeply rooted traditional beliefs lead many adolescents to be reluctant to actively discuss dysmenorrhea, let alone undergo gynecological evaluations, which directly limits the coverage and reach of early intervention programs.

To address these challenges, optimization efforts should be advanced across three core domains: First, the development of standardized clinical guidelines for adolescent dysmenorrhea prevention and control, which clearly define screening criteria, intervention pathways, and multi-sectoral collaboration mechanisms, while advocating for the inclusion of dysmenorrhea screening as a statutory component of routine adolescent health examinations; Second, the enhancement of specialized training in adolescent gynecology for frontline healthcare providers and school health personnel, the expansion of adolescent gynecology specialist clinic infrastructure, and the establishment of adolescent-friendly, confidential clinical care environments; Third, the widespread dissemination of evidence-based dysmenorrhea health information via mainstream media, public health campaigns, and community-based initiatives, to eliminate menstrual stigma and persistent cognitive misconceptions, and to foster society-wide attention to female adolescent reproductive health.

In resource-limited settings, a tiered implementation strategy is advised: (1) integrate brief dysmenorrhea questions into existing school health checks; (2) train community health workers on red-flag recognition; (3) leverage mobile health platforms for education and triage where specialist access is limited.

Preliminary modeling suggests that early dysmenorrhea intervention could reduce downstream costs associated with chronic pelvic pain management and infertility treatment, warranting health economic evaluation in diverse settings.

In summary, the systematic integration of dysmenorrhea screening into routine adolescent health examinations, school-based reproductive health education, and preconception care service systems, paired with the establishment of an integrated prevention and control model encompassing early screening, early education, early intervention, and full-cycle management, constitutes the core strategy for shifting dysmenorrhea management from passive symptomatic treatment to proactive preventive control. We urge ministries of health and education to pilot integrated dysmenorrhea screening modules within adolescent health packages, with monitoring frameworks to assess impact on school attendance, mental health, and referral completion rates. This paradigm shift not only effectively reduces the direct disease burden of dysmenorrhea, but also significantly lowers the incidence of highly prevalent reproductive-age morbidities including endometriosis, chronic pelvic pain, and infertility. This approach carries substantial practical significance for improving women's reproductive health across the lifespan and reducing overall public health expenditure.
